# Prevalence of Dyslipidemia in HIV-Positive Women with HPV Coinfection: A Preliminary Study

**DOI:** 10.1155/2021/4318423

**Published:** 2021-11-02

**Authors:** Mônika Machado de Carvalho, Karina Donato Fook, Maria José Abigail Mendes Araújo, Sulayne Janayna Araújo Guimarães, Camila Penha Abreu Souza, Carla Déa Trindade Barbosa, Ana Cléa Cutrim Diniz de Morais, Alessandra Costa de Sales Muniz, Deborah Rocha de Araújo, Maria Fernanda Bezerra Lima Bertolaccini, Ilka Kassandra Pereira Belfort, Marcelo de Souza Andrade, Sally Cristina Moutinho Monteiro

**Affiliations:** ^1^Postgraduate Student in Adult Health Program of Federal University of Maranhão-(UFMA) and Professional of Clinical Analysis and Histocompatibility of University Hospital of Federal University of Maranhão (UFMA), São Luís, Brazil; ^2^Laboratory Professional of Clinical Analysis and Histocompatibility of Universitary Hospital of Federal University of Maranhão (UFMA), São Luís, Brazil; ^3^Federal University of Maranhão (UFMA), São Luís, Brazil; ^4^Professors of Post-Graduation Adult Health Program of Federal University of Maranhão (UFMA), São Luís, Brazil

## Abstract

**Objective:**

The present study aimed to evaluate the lipid profile and atherogenic indexes in HIV-positive women with and without coinfection with human papillomavirus.

**Methods:**

Preliminary study was conducted with HIV-positive women. Laboratory tests (lipid profile, glycid profile, and atherogenic indexes) and detection of human papillomavirus (nested PCR technique using PGMY 09 and 11 primers, GP+5, and GP+6) were performed. For the analysis of the results, the data were categorized into two groups: with coinfection (HIV+/HPV+) and without coinfection (HIV+/HPV–).

**Results:**

Eighty-two HIV-positive women, aged between 35 and 49 years, participated in this study among whom 50% had HPV coinfection (HIV+/HPV+). Regarding comorbidities, there was a predominance of dyslipidemia (46.3%). The analysis of laboratory determinations and atherogenic indexes showed statistical relevance in the serum concentrations of total cholesterol (*p*=0.04), LDL cholesterol (*p*=0.03), and non-HDL cholesterol (*p*=0.04), as well as for the Castelli I index, Castelli II index, and atherogenic coefficient (*p*=0.04, 0.04, and 0.03, respectively).

**Conclusion:**

The present study demonstrated a correlation between the lipid profile and atherogenic indexes with HIV/HPV coinfection, demonstrating a possible synergy between these viruses. However, further studies in this area must be carried out.

## 1. Introduction

The human immunodeficiency virus (HIV) remains a major public health problem worldwide. It is estimated that 37.9 million people were living with HIV in 2019 and that approximately 1.7 million new cases are expected to arise worldwide [1]. In Brazil, the average number of new cases in 2018 was 17.8 per 100 thousand inhabitants. Maranhão, Legal Amazon region, is among the 11 Brazilian states that had more new cases than the national average. The city of São Luís, capital of Maranhão, presented a rate of 44.1 cases/100 thousand inhabitants, in 2018, a value 2.5 times higher than the national average, thus occupying the sixth position in the ranking of the detection rate (for 100,000 inhabitants) [2].

After the discovery of HIV, the search for treatment began, and with the advent of combined antiretroviral therapy (highly active antiretroviral therapy-HAART), significant successes were achieved [3]. However, prolonged exposure to HAART can increase the risk of metabolic complications, such as arterial hypertension (AH), dyslipidemia, and cardiovascular diseases (CVD), among others [4–6].

Before the introduction of HAART, there were reports of hypertriglyceridemia; however, after its use, new changes in lipid metabolism started to be observed, and although its etiology is not completely elucidated, dyslipidemia in people living with HIV is highly prevalent [4, 7]. In addition, adipose tissue dysfunctions, such as increased number of adipocytes and less vascularization, are common in these people [8, 9]. In this context, HIV seropositive individuals have a higher risk of developing atherosclerosis when compared to seronegative individuals and, consequently, a higher cardiovascular risk. This phenomenon has been attributed to HAART, immunodeficiency, and the inflammatory process associated with the virus [4, 6, 10].

Although atherogenesis is a multifactorial process, abnormalities in lipid metabolism are one of the main factors for its development. In general, the assessment of coronary risk is based on the determination of serum concentrations of the lipid profile, more precisely of LDL cholesterol, but scientific data have shown that this assessment is not sufficient to estimate cardiovascular risk [11, 12].

In this context, atherogenic indexes (Castelli index I and II, plasma atherogenic index, and atherogenic coefficient) have been shown to be better predictors for the risk of cardiovascular disease when compared to the absolute levels of lipids or lipoproteins [11, 12]. Kannel [13] reports that the atherogenic indexes, in the Framingham study, were more useful than the isolated assessment of the absolute levels of lipids and lipoproteins as markers of CVD risk [14].

Currently, the potential risk of developing cardiovascular disease in people with human papillomavirus (HPV) is also emphasized, due to the increased incidence of CVD in DNA-HPV-positive women. The mechanism involved in this rate is still unclear, but it has been observed that the formation of atheromatous plaque occurs earlier in these people, probably due to the constant stimulation of the immune system and systemic inflammatory process [15–18].

The study by Kuo and Fujise [16] found that HPV infections can contribute to CVD (increase the chance by 2.5 times) since viral oncoproteins bind to the tumor suppressor protein (p53) and induce its degradation, which may be associated with atherosclerosis. This association was independent of the coexisting medical conditions and traditional cardiovascular risk factors, such as smoking, high blood pressure, obesity, and dyslipidemia. Partly corroborating these findings, a cohort developed by Joo et al. [19] showed that HPV infection with high oncogenic risk was associated with an increased risk of developing CVD, especially in obese individuals with metabolic syndrome.

Therefore, coinfection between HIV and HPV viruses are linked, in addition to immunosuppression caused by HIV, with chronic systemic inflammation, which plays an important role in the development of cancer, atheroma plaque, and cardiovascular disease. In this context, knowing that it is of paramount importance to understand the biological interactions between HIV and HPV, the present study aimed to evaluate the lipid profile and atherogenic indexes in HIV-positive women with coinfection with human papillomavirus.

## 2. Methods

### 2.1. Ethical Declarations

This research was approved by the Ethics and Research Committee of the University Hospital of the Federal University of Maranhão (HU-UFMA), São Luís/MA (approval #2.776.970). Written informed consent was obtained from all women.

### 2.2. Study Population

This is a preliminary study (nonprobabilistic sample) carried out in the period between August 2018 and December 2019; a cross-sectional study was carried out in which 82 HIV-positive women, voluntarily accepted to participate in the present study; the participants were attended at two reference centers for HIV treatment (Ambulatory of Gynecology and Laboratory of Clinical Analysis and Histocompatibility of the University Hospital of the Federal University of Maranhão and Ambulatory of Patients of the Specialized Service in the Basic Health Unit of Bairro de Fátima in São Luís/Maranhão). Inclusion criteria were as follows: (1) age ≥18 years, (2) previous HIV diagnosis, and (3) being on antiretroviral therapy. Pregnant women, women with contraindications for Pap smears (e.g., current use of vaginal eggs, menstruation, and vaginal douches in the last 24 h), and women with hysterectomies were not included.

All participants answered the questionnaire about their sociodemographic data (age, educational level, sexarche, spouse, number of sexual partners, and type of sexual practice, among others), current history of the disease, continuous use of drug treatment, and life habits (e.g., use of tobacco and alcohol).

### 2.3. Biological Sample Collection

Samples of cervical swabs were collected for the detection of human papillomavirus and blood samples for the realization of the participants' glycid and lipid profiles. Conventional cytological smears were obtained with Ayre's spatula (ectocervical sample) and endocervical brush (endocervical sample), extended in a glass slide, fixed with ethanol, and stained using the Pap technique. Cytological examinations of Pap smear were reported using the 2001 Bethesda Reporting System. Venous blood samples were obtained from volunteers after a 12-hour fasting period. After collection into tubes without anticoagulant, plasma and serum were separated by centrifugation and stored in freezer at -20 degree Celsius until further analysis.

### 2.4. Laboratory Tests

The cobas® 6000 equipment, in the cobas c 501 modules, was used for the determination of fasting blood glucose (GL), total cholesterol (TC), HDL cholesterol (HDLc), and triglycerides (TG), and cobas c module was used for the measurement of serum insulin. With the data of the lipid profile, it was possible to perform the calculations of LDL cholesterol {(CT – HDLc) – (TG/5)}, non-HDL cholesterol (CT – HDLc), and atherogenic indexes: Castelli I index: CI/I (CT/HDLc ratio), Castelli II index: CI/II (LDLc/HDLc ratio), plasma atherogenic index: AIP {(logTG)/HDLc}, and atherogenic coefficient: AC {(non-HDLc/HDLc}. And with the determination of the glycidic profile was possible to calculate the TyG index (TG/GL ratio) and the HOMA-IR index (fasting glycemia x 0.0555 x fasting insulin/22.5). The HOMA and TyG indexes were used to verify possible insulin resistance (IR).

### 2.5. HPV DNA Detection

DNA extraction was performed using a cervical swab using the Biopur Mini Spin Plus Extraction Kit (Biometrix, PR, Brazil), following the protocol described in the manufacturer's user manual. For DNA-HPV detection, the Nested Polymerase Chain Reaction technique was used in a Gene Amp PCR system 9700 thermocycler (Applied Biosystems, Thermo Scientific, California, USA), with the PGMY 09 and 11 primers (amplifying sequences of 450 pb of the L1 region of the viral DNA) and GP+5 and GP+6 (amplify 190pb sequences of the L1 region of the viral DNA) [20]. The proportions of reagents used in the PCR were in accordance with that described by Nunes et al. [21]. As a positive control of the reaction, HPV-positive samples were used, and as a negative control, ultrapure water was used.

### 2.6. Statistical Analysis

The data considered categorical were presented in proportion, using the Chi-square test and Fisher's exact test. The normality of the distribution of numerical variables was assessed by the Kolmogorov–Smirnov test. For the numerical variables, Student's *t*-test was used to verify the differences between the means of the groups evaluated. The results obtained provided their distribution in two groups: with coinfection (HIV+/HPV+) and without coinfection (HIV+/HPV–). A 95% confidence interval and a significance level of *p* ≤ 0.05 were adopted for the analysis performed. All tests were applied using IBM SPSS® Statistics software version 24.0.

## 3. Results

In the present study, 82 HIV seropositive women participated, with a predominance of the 35-49-year-old age group, where 50% (n 41) were positive for DNA-HPV. Regarding the sociodemographic data, in both groups, 68.3% have up to 9 years of study, the average family income was up to one minimum wage and no condom use (51,2%). The group without coinfection had more women without fixed partners (56.1%); reported that their first relationship was between >15 years old (51.2%); that they had already become pregnant several times (73.2%) and who do not use oral contraceptives (97.5). In comparison, the coinfected group had more women with married/stable relationship (51.2%) and reported that their first relationship was between 12 and 15 years old (48.8%) and that they had already become pregnant several times (78%). There are no statistically significant results in these parameters (Table 1). The group without coinfection had more women without fixed partners (56.1%), who do not use oral contraceptives (97.5%), nonsmokers (92.7%), and nonusers of illicit drugs (95.1%). In comparison, the coinfected group reported that their first relationship was between 12 and 15 years old (51.2%) and that they had already become pregnant several times (78%) (Table 1). The most common comorbidities in HIV-positive women with HPV coinfection were dyslipidemia (46.3%) and smoking (46.3). In comparison, the group without coinfection was dyslipidemia (43.9%) and smoking (48.6). Regarding the consumption of alcoholic beverages, participants without coinfection reported making greater use of it (41.5%), which generated a statistically significant result (*p* = 0.045) when comparing the groups (Table 2).

The laboratory determinations are described in Table 3. Regarding the lipid profile of these women, it was found that 43.9% had changes in the total cholesterol concentration, 30.48% HDL cholesterol, 34.14% LDL cholesterol, and 29.26% of triglycerides, with statistical significance in the serum concentrations of total cholesterol (*p*=0.04), LDL cholesterol (*p*=0.03), and non-HDL cholesterol (*p*=0.04) when comparing the two groups. In addition, there were statistically significant values in the calculations of the Castelli I index, Castelli II index, and atherogenic coefficient (*p*=0.04, 0.04, and 0.03, respectively).

The glycidic profile was also analyzed, and 9.75% had altered fasting glycemia, and despite the glycidic profile indexes (fasting glycemia: *p*=0.36, insulin: *p*=0.85, HOMA index: *p*=0.09, and TyG: *p*=0.41) present a higher concentration in the group with coinfection, this result was not sufficiently different between the groups to generate statistically significant values (Table 3).

## 4. Discussion

The longevity of people with HIV has led to an increase in non-communicable chronic comorbidities (NCCs), including cardiovascular disease (CVD), diabetes and other metabolic conditions, renal disease, liver disease, cancers, and mental illness [4, 6]. Recently, women with a genital HPV infection were found to have an increased incidence of cardiovascular diseases (CVD), including severe cardiovascular events such as myocardial infarction and stroke [16, 19, 22].

The pathomechanisms of these relations are not yet fully understood and may significantly affect the health of a large part of the population. Accelerated atherosclerosis is assumed to play a key role in the pathophysiology of these relationships. In this context, the present study aimed to evaluate the lipid profile and atherogenic indexes in HIV-positive women with coinfection with human papillomavirus.

Although it is not a representative sample of people living with HIV from the Legal Amazon region, Maranhão/Brazil, the data are preliminary and are similar to the general epidemiological data available from people living with HIV and to other studies carried in Brazil.

With regard to socioeconomic issues, the two groups were very homogeneous, with no statistically significant differences between them; however, there are similarities with previously published research as in Ng'adwe et al. [23], where more than 50% of women did not use condoms, even though they knew the recommendations; or in Menon et al. [24], where they observed that the median of the first sexual intercourse in the group of patients with coinfection was the same (the median age was 18 years old); and in Hessol et al. [25] who observed in their study that 47% of the participants reported having had anal intercourse, a value close to that found in the present study.

Regarding comorbidities, in the present study, it can be observed that only the consumption of alcoholic beverages alone showed statistically significant values between the groups; however, for the diagnosis of comorbidity, not only isolated factors are observed, but several factors are also predisposing for CVD. Thus, dyslipidemia was present in 45.12% of the participants' results, which is an important cardiovascular risk factor.

Although HIV infection and its therapies have been associated with changes in adipose tissue and disorders of glucose and lipid metabolism that may prematurely increase the risk of cardiovascular disease [4, 6], more recent data suggest that immune activation and inflammation of chronic HIV infection also play an important role [26]. Thus, metabolic changes have been of great concern in HIV-infected adults since the introduction of antiretroviral therapy. Paradoxically, the issue of cardiovascular risk related to genital HPV infection, precancerous cervical conditions, and carcinoma represents an up-to-date topic, which has been scarcely studied.

Dyslipidemia has several origins and can have the most varied types of implications, such as cardiovascular diseases. From a physiological and clinical point of view, total cholesterol and triglycerides are among the most biologically relevant lipids for health since cholesterol is a precursor to steroid hormones, bile acids, and vitamin D [27]. It was found that approximately half of the participants had dyslipidemia, with statistical significance in the serum concentrations of total cholesterol (*p*=0.04), LDL cholesterol (*p*=0.03), and non-HDL cholesterol (*p*=0.04) when compared to the group of HIV-positive women with and without HPV coinfection. In addition, there were statistically significant values in the calculations of the Castelli I index, Castelli II index, and atherogenic coefficient. With these data, it is possible to raise the hypothesis of a possible synergy between the HIV and HPV viruses in lipid disorders and risk for cardiovascular diseases [28].

The pathophysiological mechanisms that could potentially determine the associations between HIV and HPV infection and CVD are not yet convincingly defined. In general, proatherosclerotic mechanisms related to infections may be local or systemic and direct or indirect.

The longer use of HAART and greater exposure to HIV can generate a chronic inflammatory process, which, in the long term, causes changes in the patterns of cellular behavior and metabolism of lipids and glycids [29]. And so, arterial inflammation can occur, which facilitates the formation of atheroma causing obstructions. These changes favor the development of atherosclerosis and its implications, such as myocardial infarction and peripheral vascular disease [30].

Suppressing viral replication with HAART significantly reduces immune activation for HIV seropositive individuals, but it does not eliminate it. These people have persistently higher levels of systemic inflammation when compared with HIV-negative controls both before and while receiving HAART. This is reflected by higher levels of serum biomarkers (including high-sensitivity C-reactive protein, Interleukin-6, and D-dimer) observed in HIV seropositive individuals compared with negative seronegative controls [10, 31, 32].

Between 2003 and 2006, one of the first large studies in the United States was conducted demonstrating the association between HPV, especially high-risk HPV types and an increased prevalence of self-reported diagnosis of myocardial infarction and stroke among HIV-positive women [16]. Brito et al. [22] in a recent study demonstrated that the results indicate that HPV infection might be associated with coronary artery disease (CAD) among climacteric women.

The systemic effects of these viruses include immune activation is both specific and non-specific, involving several mechanisms. The increased release of cytokines, activation, and proliferation of T-helper cells elevated the interferon-alpha, interferon-gamma, tumor necrosis factor-alpha, interleukins IL-1*β*, IL-6, IL-17, and IL-18 and decreased anti-inflammatory properties of high-density lipoprotein (HDL), reactive oxygen species formation, and procoagulant activity [18].

The present study corroborates with such CVD risk findings in women with human papillomavirus when the groups are analyzed through the lipid profile and atherogenic indices, such as Castelli I, Castelli II index, and atherogenic coefficient. The resulting effects of immune activation and chronic inflammation on the cardiovascular system are far reaching and likely explain the increased risk for cardiovascular events noted in this population. To our knowledge, this is the first study to investigate the lipid profile and atherogenic indexes in HIV-positive women with coinfection with human papillomavirus, and these results propose the hypothesis that a possible synergy between the HIV and HPV viruses into immune activation and risk factors for cardiovascular disease, although more studies are needed to investigate the mechanisms involved.

We encountered a limitation in this study that even presenting statistically significant data, it does not have a large sample size when compared to people living with HIV in the state of Maranhão. Since women living with HIV self-describe themselves and are afraid of exposure, this makes it difficult to make an estimated sample calculation. The prejudice itself is also counted as a limitation, as many women refused to sign the consent form for fear of exposure, and others gave up for fear of having to present themselves to a different group from the multiprofessional team that lives with them daily or monthly; these are the reasons for a lost sample number. However, our study analyzes the lipid profile in HIV people with HPV coinfection, a ratio that has not been carried out in previous studies, which emphasizes the importance of this. This work has practical evidence that highlights the need for studies aimed at better understanding the mechanisms of CVD in HIV and HPV infections, defining clear intervention targets, and identifying clinically relevant markers to measure response.

## 5. Conclusion

The present study demonstrated a high prevalence of HIV/HPV coinfection. The lipid profile (total cholesterol and LDL cholesterol) and the atherogenic indexes (Castelli I index, Castelli II index, and atherogenic coefficient) showed a correlation with HIV/HPV coinfection, demonstrating a possible synergy, in lipid disorders and cardiovascular risk, between the two viruses. However, further studies in this area must be carried out.

## Figures and Tables

**Table 1:** Sociodemographic characteristics of HIV-positive women with and without HPV coinfection, São Luís, Maranhão, Brazil, 2018–2019.

	HPV-positive (%) *N* = 41	HPV-negative (%) *N* = 41	*p* value	
Age			
** **18 to 34 years	29.3	22.0	0.691	
** **35 to 49 years	39.0	39.0				
** **Older than 55 years	31.7	39.0				
Schooling			
** **≤9 years	68.3	68.3	0.648	
** **>9 to 12 years	22.0	26.8				
** **>12 years	9.8	4.9				
Marital situation				
** **Married/stable relationship	51.2	43.9	0.507	
** **Single/widow	48.8	56.1				
Family income				
** **Up to 1 min wage	70.7	65.9	0.853	
** **1.1 to 3 min wages	24.4	26.8				
** **3.1 to 5 min wages	4.9	7.3				
Age of first sexual intercourse				
** **<12 years	0	0	0.825	
** **12 to 15 years	51.2	48.8				
** **>15 years	48.8	51.2				
Number of sex partners				
** **≤3	53.7	46.3	0.775	
** **4 to 6	29.3	31.7				
** **≥7	17.1	22.0				
Pregnancy				
** **Never	9.8	7.3	0.638	
** **Once	12.2	19.5				
** **Several times	78.0	73.2				
Abortions				
** **Yes	43.9	41.5	0.823	
** **No	56.1	58.5				
Condom use				
** **Yes	48.8	48.8	1.0	
** **No	51.2	51.2				
Anal sexual relationship				
** **Yes	41.5	48.8	0.506	
** **No	58.5	51.2				
Oral sexual relationship				
** **Yes	53.7	39.0	0.184	
** **No	46.6	61.0				
Same-sex sex				
** **Yes	7.5	4.9	0.624	
** **No	92.5	95.1				
Oral contraceptives				
** **Yes	5.1	2.5	0.541	
** **No	94.9	97.5				

Data are presented in proportion. Tests used: Chi-square and Fisher's exact tests. Family income is R$ 1.045,00. Source: elaborated by the authors, 2020.

**Table 2:** Comorbidities in HIV-positive women with and without HPV coinfection, São Luís, Maranhão, Brazil, 2018–2019.

	HPV-positive (%) *N* = 41	HPV-negative (%) *N* = 41	*p* value
Dyslipidemia			
** **Yes	46.3	43.9	0.40
** **No	53.7	56.1	
Arterial hypertension			
** **Yes	2.4	2.4	1.00
** **No	97.6	97.6	
Mental disorder			
** **Yes	12.2	9.8	0.70
** **No	87.8	90.2	
Smoking			
** **Yes	46.3	48.6	0.80
** **No	53.7	51.1	
Use of alcohol			
** **Yes	22.0	41.5	0.045
** **No	78.0	58.5	
Illicit drugs			
** **Yes	7.3	4.9	0.60
** **No	92.7	95.1	

Data are presented in proportion. Tests used: Chi-square and Fisher's exact tests. Source: elaborated by the authors, 2020.

**Table 3:** Laboratory results of HIV-positive women with and without HPV coinfection, São Luís, Maranhão, Brazil, 2018–2019.

	HPV-positive *N* = 41	HPV-negative *N* = 41	*p* value
Glucose fasting	87.13 ± 14.37	84.84 ± 11.46	0.36
Insulin	9.39 ± 8.47	9.8 ± 7.33	0.85
Homa-IR	2.09 ± 2.29	2.14 ± 1.85	0.9
TyG	8.60 ± 0.54	8.43 ± 1.15	0.41
Total cholesterol	195 ± 41.34	171 ± 52.08	0.04
Triglycerides	139.2 ± 73.41	138.28 ± 71.23	0.95
HDL-C	42.31 ± 13.26	44.44 ± 15.40	0.54
LDL-C	118.39 ± 35.77	98.2 ± 38.36	0.03
Non-HDL-C	119 ± 54.9	143 ± 44.5	0.04
Castelli index I	4.7 ± 1.41	4.00 ± 0.77	0.04
Castelli index II	2.89 ± 1.09	2.34 ± 0.66	0.04
Plasma atherogenic index	0.5 ± 0.25	0.49 ± 0.25	0.8
Atherogenic coefficient	2.99 ± 0.75	3.64 ± 1.38	0.03

Data are presented as mean ± standard deviation. Tests used: Student's *t*-test. Abbreviations: TyG: relationship between triglycerides and glycemia; HDL-C: high-density lipoprotein cholesterol; LDL-C: low-density lipoprotein cholesterol; and non-HDL-C: not high-density lipoprotein cholesterol. Source: elaborated by the authors, 2020.

## Data Availability

The data sets that support the conclusions of this article are included within the article.
